# Latin Dance and Working Memory: The Mediating Effects of Physical Activity Among Middle-Aged and Older Latinos

**DOI:** 10.3389/fnagi.2022.755154

**Published:** 2022-04-15

**Authors:** Susan Aguiñaga, Navin Kaushal, Guilherme M. Balbim, Robert S. Wilson, JoEllen E. Wilbur, Susan Hughes, David M. Buchner, Michael Berbaum, Edward McAuley, Priscilla M. Vásquez, Isabela G. Marques, Tianxiu Wang, David X. Marquez

**Affiliations:** ^1^Department of Kinesiology and Community Health, University of Illinois at Urbana-Champaign, Urbana, IL, United States; ^2^Department of Health Sciences, Indiana University, Indianapolis, IN, United States; ^3^Department of Physical Therapy, University of British Columbia, Vancouver, BC, Canada; ^4^Department of Neurological Sciences and Psychiatry and Behavioral Sciences, Rush Alzheimer’s Disease Center, Rush University Medical Center, Chicago, IL, United States; ^5^College of Nursing, Rush University, Chicago, IL, United States; ^6^Center for Research on Health and Aging, University of Illinois at Chicago, Chicago, IL, United States; ^7^Department of Urban Public Health, College of Science and Health, Charles R. Drew University of Medicine and Science, Los Angeles, CA, United States; ^8^Capes Foundation, Ministry of Education, Brasília, Brazil; ^9^Department of Kinesiology and Nutrition, University of Illinois at Chicago, Chicago, IL, United States

**Keywords:** cognition, exercise, disparities, dance, physical activity

## Abstract

**Background:**

Physical activity (PA) is a promising method to improve cognition among middle-aged and older adults. Latinos are at high risk for cognitive decline and engaging in low levels of PA. Culturally relevant PA interventions for middle-aged and older Latinos are critically needed to reduce risk of cognitive decline. We examined changes in cognitive performance among middle-aged and older Latinos participating in the BAILAMOS™ dance program or a health education group and compared the mediating effects of PA between group assignment and change in cognitive domains.

**Methods:**

Our 8-month randomized controlled trial tested BAILAMOS™, a 4-month Latin dance program followed by a 4-month maintenance phase. A total of 333 older Latinos aged 55+ were randomized to either BAILAMOS™, or to a health education control group. Neuropsychological tests were administered, scores were converted to z-scores, and specific domains (i.e., executive function, episodic memory, and working memory) were derived. Self-reported PA was assessed, and we reported categories of total PA, total leisure PA, and moderate-to-vigorous PA as minutes/week. A series of ANCOVAs tested changes in cognitive domains at 4 and 8 months. A mediation analysis tested the mediating effects of each PA category between group assignment and a significant change in cognition score.

**Results:**

The ANCOVAs found significant improvement in working memory scores among participants in the dance group at month 8 [*F*_(1,328)_ = 5.79, *p* = 0.017, *d* = 0.20], but not in executive functioning [*F*_(2,328)_ = 0.229, *p* = 0.80, Cohen’s *d* = 0.07] or episodic memory [*F*_(2,328)_ = 0.241, *p* = 0.78, Cohen’s *d* = 0.05]. Follow-up mediation models found that total PA mediated the relationship between group assignment and working memory, in favor of the dance group (β = 0.027, 95% CI [0.0000, 0.0705]). Similarly, total leisure PA was found to mediate this relationship [β = 0.035, 95% CI (0.0041, 0.0807)].

**Conclusion:**

A 4-month Latin dance program followed by a 4-month maintenance phase improved working memory among middle-aged and older Latinos. Improvements in working memory were mediated by participation in leisure PA. Our results support the current literature that leisure time PA influences cognition and highlight the importance of culturally relevant PA modalities for Latinos.

**Clinical Trial Registration:**

[www.ClinicalTrials.gov], identifier [NCT01988233].

## Introduction

Twelve percent of older Latinos in the U.S. are currently diagnosed with Alzheimer’s Disease (AD), and it is estimated that the number of Latinos with AD will increase by 832% by 2060 (Wu et al., 2016). Currently, there is no cure for AD; however, evidence suggests that protective factors for AD include regular physical activity (PA) (Blondell et al., 2014), cognitively and mentally stimulating leisure activities (Wilson et al., 2012), social engagement, and having a rich social network (Marioni et al., 2015). Latin dance is a particularly promising PA modality that targets these factors and is a culturally acceptable type of PA for middle-aged and older Latinos (Valenzuela et al., 2013; Marquez et al., 2014a).

Latin dance has long been part of the history, socialization, and culture of Latinos (Gonzalez et al., 1998). Dance as a PA modality for Latinos may evoke positive emotional responses that encourage PA participation in a population with low levels of PA (Arredondo et al., 2015). Latinos have cited dance as an important and desirable component to community-based programs (Larsen et al., 2015; Predovan et al., 2019). Furthermore, engaging in dance has been shown to improve or maintain cognition (Hamacher et al., 2015; Hwang and Braun, 2015; Merom et al., 2016; Predovan et al., 2019; Muiños and Ballesteros, 2021), partly due to the learning component and coordination of dancing (Voelcker-Rehage and Niemann, 2013; Rehfeld et al., 2017), the multisensory demands of the activity (Thøgersen-Ntoumani et al., 2018), the timing, synchronization and sequences of moves, and the energy expenditure associated with movement (Keogh et al., 2009). Randomized controlled trials (RCTs) have examined changes in cognitive performance among several types of dance styles including, ballroom dancing (Lazarou et al., 2017), Cha-Cha (Kim et al., 2011), and a blend of styles (e.g., line dance, jazz dance, rock’n roll, Latin, and square dance) (Hamacher et al., 2015) and have demonstrated changes in global cognition, executive function, episodic and working memory, and attention. Despite the benefits and appeal of dance, PA programs and interventions rarely implement dance programs *specifically* for Latinos, a historically excluded population at high risk of chronic disease, mobility disability (Angel et al., 2015), and cognitive impairment (Wu et al., 2016).

Given the need to address health inequities in Latinos, Marquez and colleagues created a Spanish-language, Latin dance program (BAILAMOS™—Balance and Activity In Latinos, Addressing Mobility in Older Adults) (Marquez et al., 2014a) for middle-aged and older Latinos to increase PA, thereby reducing risk of chronic diseases, mobility disability, and cognitive impairment. An initial pilot study with twelve participants demonstrated that self-reported PA increased significantly and small positive effects for executive function and speed of processing were seen in participants, all of whom received the Latin dance program (Marquez et al., 2014a). Following that small pilot, a two-group pilot trial was conducted to examine differences in cognitive performance among 57 older Latinos randomized to either the BAILAMOS™ dance program or a health education program (Marquez et al., 2017). Findings showed greater improvement in episodic memory in the dance group compared to the health education group. Balbim et al. (2021) examined the impact of the BAILAMOS™ dance program on physical activity, brain functional connectivity, and cognitive performance among 10 participants who received the program. Participation in the program led to an increase in self-reported moderate leisure-time PA, increases in brain functional connectivity, but no statistically significant changes in cognitive performance. These studies demonstrated promising findings for increasing PA, cognition, and brain function; however, these studies were limited by small sample sizes and short follow-up periods, making advanced analytical methods difficult to conduct. Therefore, the purpose of the present study was to examine the secondary outcome of changes in cognitive performance in a large sample of middle-aged and older Latinos participating in the BAILAMOS™ dance program or a health education control group at 4-months and 8-months post-intervention and compare the mediating effects of types of PA (i.e., minutes/week of total PA, minutes/week of total leisure PA, and minutes/week of moderate-to-vigorous PA), and between group assignment and change in cognitive domains.

## Materials and Methods

### Participants and Design

Study details regarding recruitment, intervention content, program delivery, and flow of participants have been previously described (Marquez et al., 2014b). Briefly, participants (*N* = 333) were low-active, at risk for disability, and cognitively healthy middle-aged and older Latinos residing in Chicago and recruited from study sites, churches with Spanish masses, health centers, presence at supermarkets, and senior fairs, to name a few. Participants were randomized into one of two conditions: (1) the 4-month, twice-weekly (1 h per session) BAILAMOS™ Latin dance program consisting of four dance styles (e.g., merengue, bachata, cha cha cha, and salsa), followed by a 4-month maintenance phase or (2) a 4-month, once weekly (2 h per session) health education condition that included curriculum on stress, My (food) Pyramid, food labels, diabetes, cancer, osteoporosis, immunizations, building a better memory, and making the most of medical appointments. The BAILAMOS™ Latin dance program encourages PA participation outside of the program to increase PA in participants’ daily lives, as the dance instructor consistently mentioned engaging in PA outside of dancing. The 4-month maintenance phase used a train the trainer model wherein indigenous leaders, participants who were sociable, proficient at dancing, and regularly attended the program were trained by the dance instructor to become dance instructors and teach the BAILAMOS™ dance program. During the 4-month maintenance phase, the indigenous leaders took attendance, led the dance classes for the participants, and continued the same structure of the initial 4-month program at different facilities. Participants in both conditions received all sessions and materials in Spanish. Assessments were conducted at baseline, the primary (month 4) and secondary (month 8) timepoints.

### Measures

#### Demographics

Information about age, gender, body mass index, education, and marital status was obtained.

#### Neuropsychological Tests

We utilized a set of neuropsychological tests (Wilson et al., 2002) which have been adapted to Spanish (Krueger et al., 2009). Seven neuropsychological tests that assess three domains (i.e., executive function, working memory, and episodic memory) that have been found to decrease with age but also to be influenced by regular PA (Kramer et al., 2006) were administered at baseline, the primary (month 4) and secondary (month 8) timepoints. Individual tests were combined to form composite scores for executive function, working memory, and episodic memory by averaging the z scores converted from each test (Wilson et al., 2002). Those z scores were computed by using the population mean and SD from the baseline measurements. Each test is described below.

##### Executive Function

Trail Making Test (TMT)-Parts A and B (Army Individual Test Battery, 1944) consist of two parts. The test requires a participant to draw lines sequentially connecting 25 encircled numbers randomly distributed on a page (Part A) and encircled numbers and letters in alternating order (Part B). The score is the time required to complete each task. Lower values reflect better performance; therefore z-scores were multiplied by −1.

Stroop Neuropsychological Screening Test: We used a short form (Wilson et al., 2005) of the Stroop Neuropsychological Screening Test (Trenerry et al., 1989) in which the participant is shown a list of color names in different ink colors, and first asked to read the words (as opposed to the color of the font), and then asked to name the color of the font (as opposed to the word itself). The scores are the number of words named correctly in 30 s minus the number of errors; and the number of colors named correctly in 30 s minus the number of errors.

Word fluency (Welsh et al., 1994) asks participants to generate as many examples as possible from two semantic categories (animals; fruits and vegetables) in separate 60-s trials. The word fluency score is the sum of the number of animals generated with the number of fruits and vegetables generated.

Symbol Digit Modalities Test (Smith, 1982) involves identifying and naming the digits which belong with consecutively presented symbols. The score is the number of digits correctly paired with symbols in 90 s.

##### Working Memory

Digit Span Test (Wechsler, 1987) has two parts. Digit strings of increasing length are read, and the participant is asked to repeat each string forward (Digit Span Forward) or backward (Digit Span Backward). The score is the number of correctly retrieved strings in each part.

Digit Ordering (Cooper et al., 1991; Wilson et al., 2005) involves reading digit strings of increasing length and the participant is asked to reorder the digits and say them in ascending order. The score is the number of correctly reordered strings.

##### Episodic Memory

Logical Memory I and II (Wechsler, 1987) has two parts. A brief story is read to the participant who is then asked to retell it immediately (I) and after a 5-min delay filled with other activities (II). The score is the number of the 25 story units recalled immediately (I) and after the delay (II).

#### Physical Activity

Self-reported PA was assessed *via* the Community Healthy Activities Model Program For Seniors (CHAMPS) Physical Activity Questionnaire for Older Adults (Stewart et al., 2001). This is a change-sensitive PA scale that assesses weekly frequency and duration of lifestyle PA (broken down into leisure time, household, occupational, transportation, and total PA) typically undertaken by older adults, reported as minutes per week. The CHAMPS has been translated into Spanish and used with older Latino adults (Rosario et al., 2008). We report categories of *total PA* (minutes of light and moderate and vigorous PA), *total leisure PA* (minutes of light and moderate and vigorous leisure PA), and *moderate-to-vigorous PA* (MVPA) as minutes/week.

### Statistical Methods

Sample size analysis, report on missing data values, and multiple imputation approaches have been previously reported (Marquez et al., 2022). The primary objective was investigated using a series of ANCOVAs to assess whether group assignment predicted change in each of the three cognitive domains (e.g., executive function, working memory, and episodic memory) at months 4 and 8. Statistically significant (*p* < 0.05) findings found at the multivariate level were followed by reporting results from the univariate tests. Changes in the primary (month 4) and secondary (month 8) time points were tested controlling for baseline cognitive values, which were treated as a covariate, with follow-up values set as the dependent variables. Age was the only demographic variable that was controlled when testing the hypotheses.

The secondary objective further investigated which PA category explained any significant between-group changes in cognitive domains at months 4 and 8 by conducting mediation analyses. Mediation models were assessed using the PROCESS macro for SPSS. Prior to analysis, all variables were standardized to z-score change values between baseline and follow-up measures based on recommended guidelines (Hayes et al., 2017). The mediation models tested if PA mediated the relationship between group assignment (dance intervention vs. control) and a significantly changed cognition score. This model was performed three times to test mediating effects for each PA category (total minutes of PA, total leisure PA, and MVPA). Statistical significance was calculated using 95% bias-corrected confidence intervals on the basis of 5,000 bootstrap samples. The effects are considered significant when the confidence intervals do not cross through zero (Preacher and Hayes, 2008; Hayes et al., 2017; Schoemann et al., 2017).

## Results

Participants were on average 64.9 (SD = 7.09) years of age with an average BMI of 31.14 (SD = 4.78). The majority of participants were female (84.4%), with 8.38 (SD = 4.01) years of education, and 48.9% were married. Age was statistically different (0.050) as the significance level was defined as <0.05 and was controlled when conducting hypotheses tests on any baseline or demographic variables including baseline total PA, total leisure PA, and moderate-to-vigorous PA. For cognitive performance scores, there were significant between-group differences for Trail Making Test A and Stroop CW (Table 1).

**TABLE 1:** Demographic characteristics of the sample.

Baseline characteristics	Control condition (*N* = 166)	Experimental condition (*N* = 167)	*p*
	Mean (SD)	Mean (SD)	
Age	65.67 (7.68)	64.14 (6.39)	0.05
Female	141	140	0.78
BMI	30.84 (5.05)	31.47 (4.54)	0.19
Years of education	8.40 (4.10)	8.35 (3.91)	0.91
Marriage/Common Law	65	98	0.92
**CHAMPS**			
Total PA	695.06 (438.54)	717.16 (533.340)	0.68
Total Leisure-time PA	341.75 (334.56)	339.79 (411.49)	0.96
Moderate-to-vigorous PA	148.73 (229.87)	169.21 (322.92)	0.51
**Cognitive tests**			
TMT-A	54.4 (27.25)	48.41 (22.88)	0.03
TMT-B	191.11 (86.94)	177.22 (85.40)	0.15
Stroop-C	51.56 (13.85)	53.93 (14.65)	0.13
Stroop-CW	17.76 (6.79)	20.13 (8.59)	0.01
Word fluency	33.87 (8.38)	34.46 (8.24)	0.52
SDMT	30.83 (13.25)	32.32 (12.12)	0.29
Digit span forward	5.03 (1.70)	4.91 (1.76)	0.55
Digit span backward	3.76 (1.71)	3.74 (1.64)	0.92
Digit ordering	5.27 (1.95)	5.55 (1.89)	0.20
Logical memory-immediate	9.18 (3.76)	9.37 (3.63)	0.64
Logical memory-delayed	7.80 (3.63)	7.80 (3.69)	0.99

*Abbreviations: CHAMPS, Community Healthy Activities Model Program For Seniors; TMT, Trail Making Test; SDMT, Symbol Digit Modalities Test.*

A series of repeated measures ANCOVA were performed to test between group differences for each cognitive domain with age as a covariate (Table 2). There was a significant group difference in working memory between the two groups [Wilk’s λ = 0.98, *F*_(2,328)_ = 3.13, *p* = 0.045]. Follow-up univariate tests found no significant between-group differences at month 4 [*F*_(1,328)_ = 0.392, *p* = 0.532, *d* = 0.13], but the experimental dance group demonstrated a significant improvement in working memory at month 8 of the study [*F*_(1,328)_ = 5.79, *p* = 0.017, *d* = 0.20]. Multivariate analyses that tested change in executive functioning [Wilk’s λ = 0.99, *F*_(2,328)_ = 0.229, *p* = 0.80] and episodic memory [Wilk’s λ = 0.99, *F*_(2,328)_ = 0.241, *p* = 0.78] were not significant between the two groups at 4 and 8 months.

**TABLE 2:** Means and standard deviations of cognitive domain scores by study condition, with effect sizes for within and between condition differences.

Constructs	Condition E (*N* = 167) C (*N* = 166)	Baseline *z-score*	Month 4 *z-score*	Month 8 *z-score*	Cohen’s *d* within condition (Month 4)	Cohen’s *d* within condition (Month 8)	Cohen’s *d* between condition (Month 4)	Cohen’s *d* between condition (Month 8)
**Cognitive Domain**								
Working memory	E	0.013	0.153	0.118	0.17	0.24	0.16	0.20
Working memory	C	0.016	−0.120	−0.090	0.02	0.04		
Episodic memory	E	0.015	0.061	0.055	0.27	0.44	−0.03	0.05
Episodic memory	C	0.012	−0.083	−0.098	0.30	0.39		
Executive functioning	E	−0.069	0.106	0.101	0.00	0.09	0.08	0.07
Executive functioning	C	0.072	−0.111	−0.110	−0.07	0.01		

### Mediation Analysis

**FIGURE 1:**
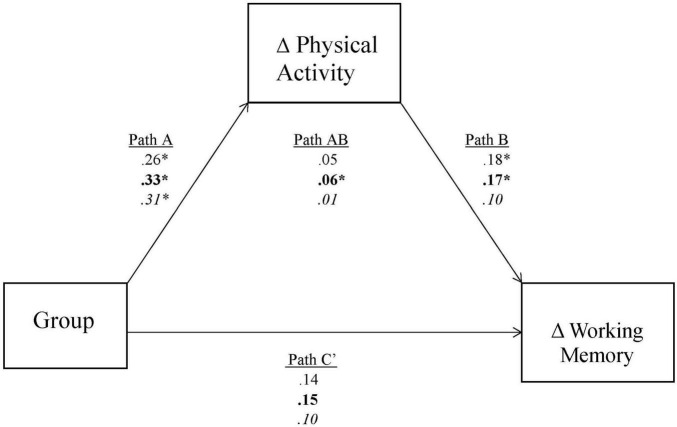
Mediation model. Coefficients in standard font, bolded font, and italics represent total physical activity time, total leisure time physical activity, and moderate-to-vigorous physical activity, respectively. *Significance at *p* < 0.05. Group compares experimental vs. control condition.

The significant findings of working memory at month 8 were further investigated with mediation tests (Figure 1). The first mediation model found that group assignment (Path A) predicted total PA time (β = 0.23, 95% CI [0.0174, 0.4463], *p* = 0.034) in favor of the dance group. Total PA time in turn (Path B), predicted higher working memory scores (β = 0.12, 95% CI [0.0098, 0.2247], *p* = 0.032). Indirect path analysis revealed total PA time mediated the relationship between group assignment and working memory (β = 0.027, 95% CI [0.0000, 0.0705]) and the direct pathway (Path C’) crossed through zero (β = 0.10, 95% CI [−0.1134, 0.3152]), thus satisfying the requirements for complete mediation.

The second mediation model found group assignment predicted total leisure PA (Path A) (β = 0.25, 95% CI [0.0397, 0.4593], *p* = 0.025), and total leisure PA predicted working memory (Path B) (β = 0.14, 95% CI [0.0367, 0.2510], *p* = 0.009) in favor of the dance group. The indirect path (Path AB) mediated the relationship between group assignment and working memory (β = 0.035, 95% CI [0.0041, 0.0807]), while the direct pathway from group assignment to working memory revealed the 95% CI to cross through zero (β = 0.11, 95% CI [−0.1048, 0.3227]), also supporting a mediating effect.

Finally, for the third mediation model, group assignment predicted MVPA (Path A) (β = 0.42, 95% CI [0.1248, 0.7088], *p* = 0.054); however, MVPA did not predict working memory (Path B) (β = 0.02, 95% CI [−0.1244, 0.1585], *p* = 0.812). Confidence intervals of the direct pathway (Path C’) (β = 0.31, 95% CI [−0.0203, 0.6035]) did not cross through zero, and indirect effects (Path AB) crossed through zero (β = 0.01, 95% CI [−0.0392, 0.0679]), denoting non- mediated effect.

## Discussion

The present study examined whether participation in the BAILAMOS™ dance program improved cognitive performance compared to a health education control group and investigated which PA category predicted cognitive changes among middle-aged and older Latinos over eight months. The study showed that participants who were randomized to the 4-month Latin dance program followed by a 4-month maintenance phase improved in working memory compared to participants in the health education control group. Findings indicate that improvements in working memory were mediated by participation in total PA and total leisure PA at 8 months only. While effect sizes (Cohen’s *d*) were modest in this study, small effect sizes are typically found in RCTs in older adults (Erickson et al., 2019). These findings support the current literature on the influence of leisure PA and total PA on cognitive performance and highlight the importance of culturally appropriate PA modalities for middle-aged and older Latinos.

The between-groups analysis between the two groups at 4-months found no differences in any of the cognitive measures, which is similar to findings from a small pilot examining the BAILAMOS™ dance program in older Latinos (Balbim et al., 2021). However, our study found improved working memory at month 8 in the dance group. This finding is in line with the literature demonstrating that longer dance interventions (>4 months) are needed to detect changes in cognitive performance. For example, Fausto and colleagues found that African American older adults who received a 5-month cardio-dance program consisting of dance routines and steps from hip-hop, merengue, samba, cumbia, and salsa improved attention compared to a propensity-matched control group (Fausto et al., 2021). Another study by Lazarou et al. (2017) reported that participants in a 10-month international ballroom dancing program improved in global cognition, reaction time, visuospatial skills, selective attention, and attentional switching. Our study found an improvement in working memory, and this finding is especially pertinent because working memory decline has been recognized as one of the primary contributing factors to cognitive impairment in older adults (Park et al., 2002). Therefore, Latin dance could be an important strategy to improve or maintain working memory among middle-aged and older Latinos, a population at high risk of cognitive decline.

Our findings demonstrated that changes in working memory at 8 months were mediated by total PA and total leisure PA. This finding suggests that PA categories may function as a mechanism to improve working memory. Studies have suggested that different types of PA may determine the extent to which PA impacts cognition (Quigley et al., 2020). For example, Quigley et al. (2020) concluded that single PA modes are effective in inducing improvements in cognition among older adults, but combined PA modes may offer magnified cognitive benefits. The advantage of dancing is that it is a PA mode that embeds aerobic PA and neuromotor PA (i.e., balance and proprioceptive training). Furthermore, participants in the Latin dance group were provided with a PA modality that facilitated an increase in their total PA and functioned as a leisure type of PA.

Changes in working memory at 8 months may have been due to several reasons. First, dancing poses a high demand on attention and memory for individuals. Participants initially learned four dance styles. During the 4-month maintenance phase, four additional dance moves were added to each dance style. Participants were able to practice what they learned during the maintenance phase, therefore the process of recalling steps and learning new dance steps may be due to the working memory process known as chunking information (Tulving and Craik, 2000). Participants in the dance intervention did this through the process of combining dance steps into sequences. For example, participants learned the side basic, back basic, and backside basic dance steps independently, which then were chunked into what is known to be the salsa dance style. Similar to the way the brain manipulates information into chunks, here we see the same process happening with the learning of different Latin dance styles, which helps to explain the improvement in working memory scores. Second, participants continued the dance program for an additional 4 months (maintenance phase). Indigenous leaders in the treatment group received training on how to continue the dance program without a professional instructor during the maintenance phase while the health education group stopped meeting. Therefore, with the train the trainer model, participants were provided the tools and training necessary to continue leading the program. Furthermore, dance participants may have felt empowered to implement strategies learned to overcome PA barriers, strengthened social ties, and continue engaging in a cognitively stimulating activity (e.g., dance). This study highlights the importance of implementing dance as a PA modality to increase PA, thereby, improving working memory and underscores how imperative it is to provide tools and training to create sustainable community programs led by community members.

Our study had several strengths including a large sample of community dwelling older, Spanish-speaking Latinos, a group that is rarely included in large RCTs, but has a high level of need for health-related interventions. The study also included a culturally relevant PA intervention (i.e., dance) with a 4-month maintenance phase embedded within the program. The study also had several limitations. First, PA was self-reported; thus, PA may be over- or under-estimated. Second, participants resided in Chicago and a majority identified as Mexican American, thereby limiting generalizability of findings to other geographic areas and other Latino subgroups. For the cognitive outcomes, improvements in cognitive performance should be interpreted with caution considering potential practice effects; however, the small changes observed in the control group suggest that practice effects, if any, are small. Also, no further follow-up was conducted to determine whether improvement in working memory were maintained. Lastly, the *a priori* sample size calculation of the trial was not performed specifically for the mediation analyses, but rather based on the primary outcome of changes in PA.

In sum, this study underscores the importance of creating and delivering culturally relevant, community-based Latin dance programs that promote total PA and function as a leisure time PA for Latinos. Furthermore, implementing maintenance phases to create more sustainable programs and empowering community members to lead programs is critical to create a culture of health. Future studies may consider including longer follow up periods to determine the duration of dance intervention effect on cognition.

## Data Availability Statement

The raw data supporting the conclusions of this article will be made available by the authors, without undue reservation.

## Ethics Statement

The studies involving human participants were reviewed and approved by University of Illinois at Chicago. The patients/participants provided their written informed consent to participate in this study.

## Author Contributions

SA wrote the manuscript with feedback and guidance from NK, GB, and DM. NK conducted the statistical analysis. RW, JW, SH, DB, MB, EM, PV, IM, and TW provided critical feedback. All authors contributed to the final version of the manuscript.

## Author Disclaimer

The content is solely the responsibility of the authors and does not necessarily represent the official views of the National Center for Advancing Translational Sciences or the National Institutes of Health.

## Conflict of Interest

The authors declare that the research was conducted in the absence of any commercial or financial relationships that could be construed as a potential conflict of interest.

## Publisher’s Note

All claims expressed in this article are solely those of the authors and do not necessarily represent those of their affiliated organizations, or those of the publisher, the editors and the reviewers. Any product that may be evaluated in this article, or claim that may be made by its manufacturer, is not guaranteed or endorsed by the publisher.
